# Mobile resistome of human gut and pathogen drives anthropogenic bloom of antibiotic resistance

**DOI:** 10.1186/s40168-019-0774-7

**Published:** 2020-01-07

**Authors:** Kihyun Lee, Dae-Wi Kim, Do-Hoon Lee, Yong-Seok Kim, Ji-Hye Bu, Ju-Hee Cha, Cung Nawl Thawng, Eun-Mi Hwang, Hoon Je Seong, Woo Jun Sul, Elizabeth M. H. Wellington, Christopher Quince, Chang-Jun Cha

**Affiliations:** 1 Department of Systems Biotechnology and Center for Antibiotic Resistome, Chung-Ang University, Anseong, 17546 Republic of Korea; 2 School of Life Sciences, University of Warwick, Coventry, CV4 7AL UK; 3 Warwick Medical School, University of Warwick, Coventry, CV4 7AL UK

**Keywords:** Antibiotic resistance, Antibiotic resistance gene, Resistome, Mobile genetic element, Horizontal gene transfer, Human gut microbiome, Pathogen, Transmission, Metagenome, Bacterial genome

## Abstract

**Background:**

The impact of human activities on the environmental resistome has been documented in many studies, but there remains the controversial question of whether the increased antibiotic resistance observed in anthropogenically impacted environments is just a result of contamination by resistant fecal microbes or is mediated by indigenous environmental organisms. Here, to determine exactly how anthropogenic influences shape the environmental resistome, we resolved the microbiome, resistome, and mobilome of the planktonic microbial communities along a single river, the Han, which spans a gradient of human activities.

**Results:**

The bloom of antibiotic resistance genes (ARGs) was evident in the downstream regions and distinct successional dynamics of the river resistome occurred across the spatial continuum. We identified a number of widespread ARG sequences shared between the river, human gut, and pathogenic bacteria. These human-related ARGs were largely associated with mobile genetic elements rather than particular gut taxa and mainly responsible for anthropogenically driven bloom of the downstream river resistome. Furthermore, both sequence- and phenotype-based analyses revealed environmental relatives of clinically important proteobacteria as major carriers of these ARGs.

**Conclusions:**

Our results demonstrate a more nuanced view of the impact of anthropogenic activities on the river resistome: fecal contamination is present and allows the transmission of ARGs to the environmental resistome, but these mobile genes rather than resistant fecal bacteria proliferate in environmental relatives of their original hosts.

Video abstract.

## Background

Over the past decades, the incidence of bacterial infections that are difficult to treat with conventional antibiotics has increased [1, 2]. The evolution of drug resistance in such pathogens is driven by both mutations on chromosomal loci and the acquisition of antibiotic resistance genes (ARGs) associated with mobile genetic elements (MGEs) [3, 4]. Since horizontal gene transfer (HGT) among bacteria occurs between different clones, taxa, and habitats [5–7], the evolutionary paths to antibiotic resistance via the acquisition of ARGs could be far more complex than those involving mutation-based resistance.

The evolutionary and ecological relationships among ARGs from environmental and clinical bacteria have been demonstrated by many studies using genomics- and metagenomics-based approaches [8, 9]. In addition, plasmid-mediated ARGs that have spread globally in recent decades, such as *qnrA*, *bla*_CTX-M_, and *mcr*-*1*, have been traced to environmental and animal origins, emphasizing the ongoing dissemination of ARGs across bacterial habitats [10–12]. In this context, the environmental microbiome is now recognized as a reservoir of ARGs observed in the clinical setting, and there is a growing appreciation for the use of integrative strategies, such as the so called “one-health approach”, in the sectors of human, animal, and environmental research to better understand the distribution and transmission of ARGs [13].

Antibiotic resistance has been shown to be ubiquitous in the environment [14]. Meta-analyses of microbiomes from various habitats such as gut, soil, and water have shown that different ecological niches contain distinct ARG contents at varying abundances [15]. Variations in ARG composition were shown to be structured by phylogenetic compositions of microbial communities [16]. Less addressed, yet critical to practical issues, is the evaluation of how human activities influence the environmental resistome and how clinically relevant ARGs are related to environmental ARGs. To address these questions, geographically distinct samples spanning various levels of anthropogenic pressure need to be explored. Dynamic changes within the river ecosystem can be viewed as a continuous succession of microbial communities along a spatial continuum [17], as human activities exerted variable impacts on river microbial communities [18]. Previous studies have demonstrated that urban inputs have led to increases in ARGs, class 1 integrons, and resistant bacterial isolates in river microbial communities [19, 20]. The Han River is a continuous aquatic ecosystem that flows across the Korean peninsula, originating in the pristine mountainous regions of the east and passing through the metropolitan city Seoul before reaching the estuary facing the Yellow Sea. The geographical setting of the Han River involves a steep human population density gradient, enabling the systematic evaluation of anthropogenic influences on the environmental resistome.

**Fig. 1:**
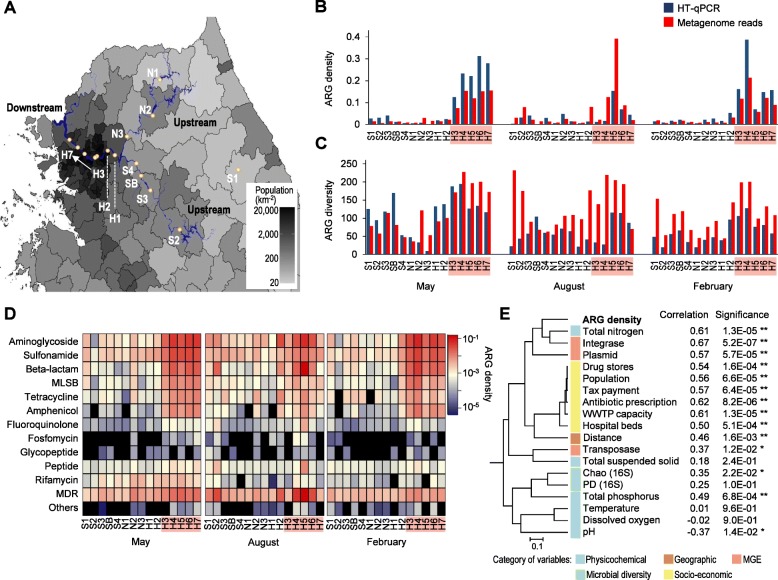
Resistome profiles of the Han River based on metagenome sequencing and high-throughput qPCR (HT-qPCR) analyses. **a** Sampling sites surveyed in this study shown on a map of the Han River (blue) along with human population density. **b** ARG density estimated from shotgun metagenomes and HT-qPCR. For metagenome reads, reads per kilobase of sequence per million mapped reads (RPKM) values of ARGs were normalized by the average RPKM of 40 single-copy genes (SCGs). For HT-qPCR data, copy numbers of targeted ARGs were normalized by those of bacterial 16S rRNA genes. **c** ARG diversity estimated by Chao richness index. The Chao index of each sample corresponds to the estimated number of ARGs present in the sample. **d** Heatmaps of ARG density distribution throughout the sampling sites according to antibiotic class. **e** Dendrogram of various sample parameters based on Pearson’s correlation distance matrix. The correlation with ARG density is shown to the right of each variable. ^*^P < 0.05, ^**^P < 0.01. Sample names corresponding to the “downstream” regions are highlighted with a red background in (**b**–**d**).

In the present study, we surveyed the microbiome, resistome, and mobilome of the planktonic microbial communities of the Han River using integrative analyses involving culture-dependent and -independent methods. This study is unique in that our samples spanned the whole length of the river, encompassing a gradient of anthropogenic impacts, and the analyses were conducted over three different seasons (Fig. 1a). Through this integrative approach, we present an in-depth characterization of the dynamics of the river resistome, driven by anthropogenic influences.

## Results

### Bloom of ARGs in the downstream Han River

We defined sampling sites H3–H7 as “downstream” regions of the Han River, as the border of the Seoul metropolitan area is located between sites H2 and H3 (Fig. 1). In Han River samples collected over three different seasons, we repeatedly observed sharp increases in ARG density in the downstream regions, where the population density is high (Fig. 1a, b). The overall ARG density per 16S rRNA gene copy, which was estimated by high-throughput quantitative PCR (HT-qPCR) array, was 2.0- to 16.0-fold higher in the downstream samples than in the upstream samples (Wilcoxon rank-sum test, *P* = 5.1 × 10^−7^; Fig. 1b). Shotgun metagenomics analysis also revealed a similar trend in ARG density per the average read depth of single-copy genes (SCGs) in each metagenome data set, with 4.8- to 10.9-fold increases in density downstream (Wilcoxon rank-sum test, *P* = 1.6 × 10^−8^; Fig. 1b). The diversity of ARGs was also greater in the downstream samples than in the upstream samples (Wilcoxon rank-sum test, *P* = 5.3 × 10^−5^ for metagenome data and *P* = 7.5 × 10^−3^ for HT-qPCR data; Fig. 1c). Bloom of ARGs in the downstream areas involved the notable enrichment of aminoglycoside, sulfonamide, β-lactam, macrolide-lincosamide-streptogramin B (MLSB), tetracycline, and amphenicol resistance genes, leading to characteristic resistome profiles in the downstream Han River (Fig. 1d).

Correlations between the ARG density and microbiological, genetic, physicochemical, geographic, and socio-economic parameters were evaluated using Pearson’s and Spearman’s correlation tests. Geographic distance, total nitrogen, and a series of socio-economic parameters, such as population density, tax payment, capacity of wastewater treatment plants, the amount of prescribed antibiotics, and numbers of drug stores and hospital beds, showed strong correlations with ARG density (Fig. 1e, Additional file 1: Figure S1 and Table S1). ARG density was also significantly correlated with the abundance of MGEs, particularly that of integrase. In contrast, bacterial diversity, temperature, dissolved oxygen, and pH appeared to be correlated with each other but exhibited little correlation with ARG density.

### Comparison of the compositions of ARGs, bacterial communities, functional genes, and fecal indicators between upstream and downstream regions

**Fig. 2:**
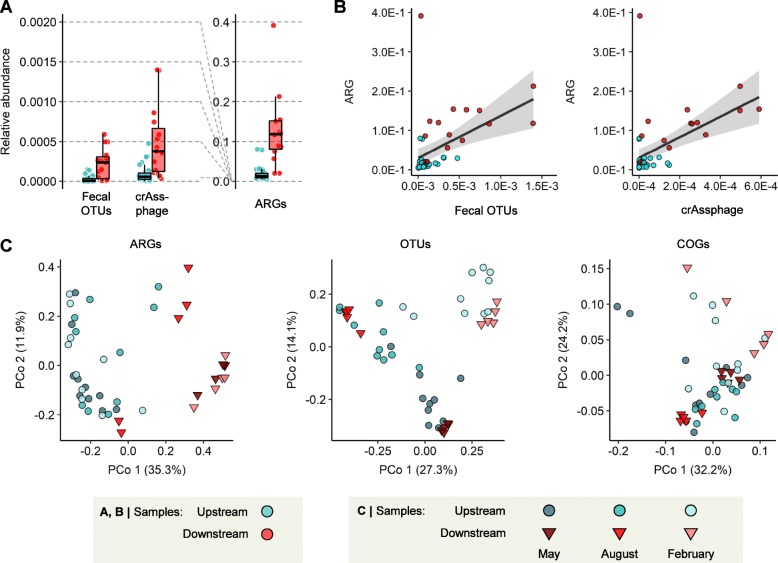
Comparison of the compositions of fecal indicators, ARGs, OTUs, and functional genes between upstream and downstream. **a** Relative abundances of human fecal indicators compared to that of ARGs. The abundance of 27 representative human fecal OTUs was estimated based on the proportions of 16S rRNA gene amplicon reads. The abundance of crAssphage was estimated based on the depth of aligned metagenome reads and normalized by the average read depth of SCGs. **b** Correlation between abundance of ARG and abundance of human fecal indicators. **c** β-Diversity of the compositions of ARGs, OTUs, and functional genes of Han River samples. Plot coordinates were determined by principal coordinate analysis of Bray–Curtis dissimilarity matrix, based on the ARG compositions analyzed from metagenome reads (left), OTU compositions from 16S rRNA gene amplicon data (middle), and COG compositions analyzed from metagenome contigs (right).

To evaluate how much fecal pollution as a measure of human influence was associated with the increase in ARGs downstream, we compared the relative abundances of the representative fecal operational taxonomic units (OTUs) and fecal indicator crAssphage [19] between upstream and downstream regions. The top 27 most abundant OTUs among the human distal gut bacterial communities in the OTU table of the Earth Microbiome Project [21], comprising 51% of total OTUs from the human gut samples, were selected as representative fecal bacteria (Additional file 1: Table S2). The relative abundances of both of these fecal indicators increased in the downstream regions (Wilcoxon rank-sum test, *P* = 2.7 × 10^−5^ for fecal OTUs and *P* = 6.8 × 10^−5^ for crAssphage; Fig. 2a) and were correlated with ARG abundance (Linear regression, *R*^*2*^ = 0.21 and *P* = 9.7 × 10^−4^ for fecal OTUs, *R*^2^ = 0.26 and *P* = 2.2 × 10^−4^ for crAssphage; Fig. 2b), indicating the influence of fecal pollution on the downstream resistome. However, the relative abundances of these fecal indicators in the downstream regions were still low, compared to that of ARGs (Fig. 2a), suggesting that the ARG bloom in the downstream regions could not be accounted solely by the input of fecal microbes.

β-Diversity analysis revealed a strong geographic contrast between upstream and downstream sites in the composition of ARGs (analysis of similarities (ANOSIM), *R* = 0.714 and *P* = 0.001), but not in the bacterial community composition (16S rRNA gene OTUs; ANOSIM, *R* = 0.075 and *P* = 0.032) or functional composition (clusters of orthologous genes (COGs); ANOSIM, *R* = 0.132 and *P* = 0.041) (Fig. 2c). This geographic contrast was apparent in the compositions of aminoglycoside, β-lactam and MLSB resistance genes. In contrast, seasonal variation was most evident in the bacterial community composition (ANOSIM, *R* = 0.735 and *P* = 0.001; Fig. 2c). Our observation that ARGs exhibited a markedly stronger geographic contrast between upstream and downstream sites than OTUs and COGs suggests that downstream human activities have a greater influence on the resistome structure than on the community structure.

### Dynamics of the River resistome along the spatial continuum

**Fig. 3:**
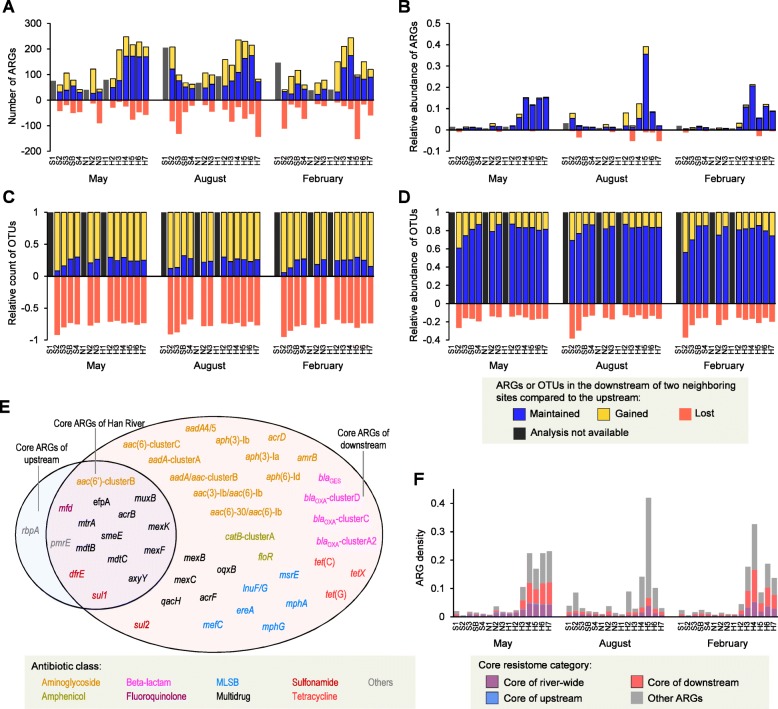
Spatial succession of ARGs and OTUs and core resistome of the Han River. **a** Number of ARGs (clustered at 99% identity) maintained, gained, and lost in the downstream member of a pair of neighboring sites compared to the upstream member. **b** Relative abundance of ARGs maintained, gained, and lost in the downstream member of a pair of neighboring sites. **c** Unweighted proportion of OTUs maintained, gained, and lost in the downstream member of a pair of neighboring sites. **d** Weighted proportion of OTUs maintained, gained, and lost in the downstream member of a pair of neighboring sites. **e** List of ARGs belonging to the core resistomes of the Han River, the downstream sites, and the upstream sites. The core resistome was defined as ARGs detected in 90% or more of samples. **f** Relative abundances of core ARGs in the river samples. ARG density was imported from data shown in Fig. 1b.

The succession of ARGs and bacterial OTUs was characterized along the spatial continuum, with a specific focus on retention, gain, and loss occurring from upstream to downstream. For this analysis, reference ARGs clustered at 99% identity were used to obtain a better resolution. Although dynamic changes in ARGs could be observed between pairs of neighboring sites (Fig. 3a), gains and losses of ARGs were minor contributors across the spatial succession of ARGs throughout the river when their abundances were considered (Fig. 3b). The proportions of ARGs remaining in the downstream member of a pair of sites for comparison were shown to be relatively constant according to a distance-decay curve (Additional file 1: Figure S2a), whereas the proportions of ARGs newly appearing in the downstream samples increased according to the distance between samples (Additional file 1: Figure S2b). In contrast, the bacterial OTUs exhibited more dynamic changes (gains and losses) than the ARGs (Fig. 3c, d). OTUs shared between two sites displayed constant distance-dependent decay patterns (Additional file 1: Figure S2c), while the proportions of OTUs newly appearing in the downstream samples increased slightly according to distance (Additional file 1: Figure S2d). These results indicate distinct successional dynamics of the river microbiome and resistome across the spatial continuum.

To characterize the ARG components that were stable throughout the river, core ARGs were defined as ARGs detected in 90% or more of the samples and were determined river-wide, as well as among the downstream and upstream sample sets (Fig. 3e). River-wide core ARGs consisted of genes encoding mutation frequency decline protein (*mfd*), aminoglycoside 6*'*-*N*-acetyltransferase (*aac*(*6'*)-clusterB), dihydropteroate synthase (*sul1*), dihydrofolate reductase (*dfrE*), UDP-glucose 6-dehydrogenase (*pmrE*), and multi-drug efflux pumps. The downstream core resistome was substantially larger than the river-wide core resistome and included a wide range of genes conferring resistance against aminoglycoside, β-lactam, tetracycline, amphenicol, and MLSB. The bloom of ARGs in the downstream samples from May and February were largely driven by these downstream core ARGs in the samples (Fig. 3f).

### ARGs associated with MGEs and HGT

In the Han River metagenomes, the prevalence of class 1 integron integrases (*intI1*) and plasmid-like contigs increased dramatically in the downstream samples (Additional file 1: Figure S3a, c). Transposases were highly abundant throughout the river, without significant enrichment in the downstream samples (Additional file 1: Figure S3b). At the metagenome contig level, ARGs were generally found to co-occur with MGEs on the same contigs at a higher frequency than COGs (Additional file 1: Figure S4a). For both COGs and ARGs, the frequency of co-occurrence with MGEs increased in the downstream samples (Additional file 1: Figure S4b). Some ARGs occurred with MGEs at an extremely high frequency (> 75%), including the GES and OXA types of β-lactamase genes, sulfonamide resistance genes (*sul*), tetracycline resistance genes (*tet*), macrolide phosphotransferase gene (*mphD*), chloramphenicol acetyltransferase gene (*catB*), and various aminoglycoside inactivation genes (Additional file 1: Figure S4c).

Based on the hypothesis that the presence of nearly identical gene sequences (≥ 99% sequence identity) in metagenome contigs or genomes assigned to different taxa at the family level is indicative of HGT between taxa [22, 23], we analyzed the HGT network of ARGs from the river metagenomes and compared it to that reconstructed from publicly available genomes of human pathogens. The HGT network from pathogen genomes revealed two subsets of mobile ARGs: set A, including ARGS shared among Proteobacteria and Actinobacteria, and set B, including those shared among Firmicutes (Additional file 1: Figure S5a). The HGT network from the river metagenomes identified *Enterobacteriaceae*, *Moraxellaceae*, and *Pseudomonadaceae* as the major hosts of horizontally transferred ARGs (Additional file 1: Figure S5b). ARGs included in the river HGT network significantly overlapped with set A, but not set B, of the pathogen HGT network (Additional file 1: Figure S5c). In both networks, the *sul1* gene showed the broadest taxonomic range (Additional file 1: Figure S5d), indicating the prevalence of class 1 integrons in a wide range of taxa [24].

### Comparison of river resistome with human gut and pathogen resistomes

To evaluate the correlation between the river resistome and the human-related resistome, we compared our river metagenome data with selected human gut metagenome data of various geographic origins [25] and human pathogen genome data available at the Pathosystems Resource Integration Center (PATRIC) database [26]. The overall ARG compositions of the river samples were clearly different from those of the human gut microbiota (Additional file 1: Figure S6a). However, the downstream river samples showed relatively higher similarities to the human gut samples than the upstream ones (*t* test, *P* < 0.001; Additional file 1: Figure S6b). Moreover, the downstream samples were more similar to Korean gut samples than they were to non-Korean ones (*t* test, *P* < 0.001; Additional file 1: Figure S6b), whereas there was no such pattern among the upstream samples.

**Fig. 4:**
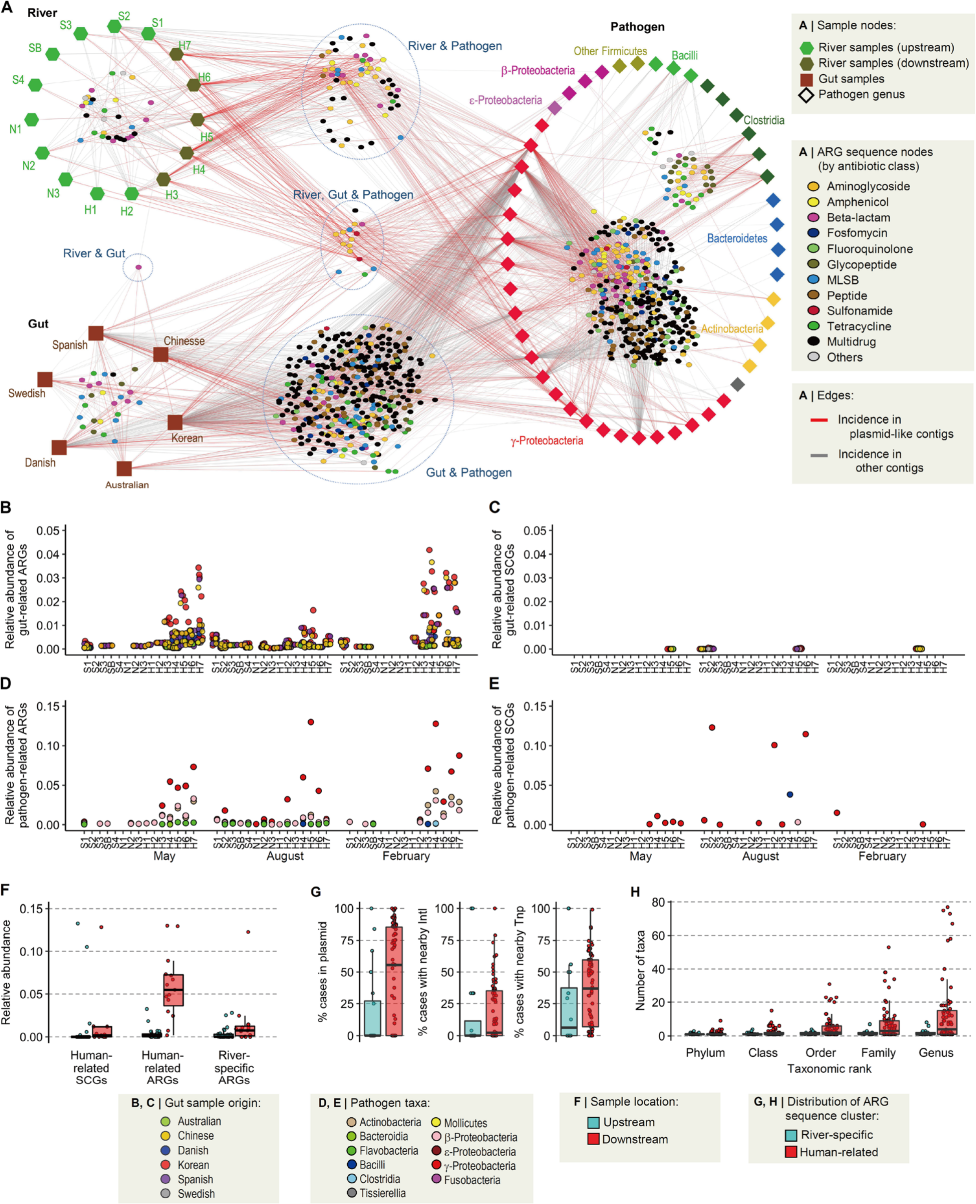
Association of river resistome with human-related ARGs. **a** Network of shared ARGs among river, human gut, and pathogen resistomes. Two types of nodes are present in the network: sample nodes and ARG sequence nodes. Each sample node represents a set of metagenome data from the same site (river metagenomes) or the same country (gut metagenomes) or a set of pathogen genomes in the same genus. Each ARG sequence node represents a unique ARG with 99% nucleotide identity. ARG nodes that contain sequences from only a single sample node were removed from the network. Edges were created between ARG sequence nodes and sample nodes, with different colors indicating the incidence of plasmid-like contigs. Sample nodes of pathogen genomes are shown in different colors according to bacterial taxa. **b** Relative abundances of gut-related ARGs in the river metagenomes. **c** Relative abundances of gut-related SCGs in the river metagenomes. **d** Relative abundances of pathogen-related ARGs in the river metagenomes. **e** Relative abundances of pathogen-related SCGs in the river metagenomes. **f** Comparison of relative abundances of human-related SCGs, human-related ARGs, and river-specific ARGs between upstream and downstream. **g** Frequency of human-related and river-specific ARGs in the context of mobile genetic elements (MGEs) in the genome database. ARGs were considered to be in an MGE context, when detected in plasmid-like contigs or within 50 kb from integrases (IntI) or transposases (Tnp). **h** The number of taxa in the genome database in which human-related and river-specific ARGs were detected.

The presence of shared ARG sequences among the river, gut, and pathogen data sets was examined based on the clustering of nearly full-length ARG sequences (480 river ARGs, 1805 gut ARGs, and 361,291 pathogen ARGs) using a 99% sequence identity cut-off. Of the resulting 9567 ARG sequence clusters, 161 clusters contained river ARGs, 54% of which contained river ARGs only (termed river-specific ARGs.) The remaining 46% contained river ARGs and pathogen and/or gut ARGs, hereafter referred to as pathogen-related ARGs and gut-related ARGs, respectively, or collectively as human-related ARGs. Human-related ARGs in the river were predominantly those related to pathogens belonging to γ-proteobacteria and were more prevalent in the downstream samples than in the upstream samples (Fig. 4a).

Interestingly, SCGs, which are relatively reliable organism-level phylogenetic markers, were not shared much between the river and gut metagenomes or between the river metagenomes and pathogen genomes, whereas ARGs were extensively shared, especially in the downstream regions (Fig. 4b–e). There was little correlation between the relative abundances of gut-related ARGs and gut-related SCGs in river samples or between the relative abundances of pathogen-related ARGs and pathogen-related SCGs. These trends suggest that ARGs, but not antibiotic-resistant bacteria (ARB), are extensively shared between the data sets. In addition, these human-related ARGs increased much more dramatically (Wilcoxon rank-sum test, *P* = 5.28 × 10^−7^ and effect size = 1.76) than human-related SCGs (*P* = 3.29 × 10^−3^ and effect size = 0.43) in the downstream regions (Fig. 4f). While human-related ARGs and river-specific ARGs exhibited similar abundances in the upstream samples, human-related ARGs were 16.8-fold more prevalent in the downstream samples than in the upstream samples, and river-specific ARGs were 4.6-fold more prevalent in the downstream samples than in the upstream samples (Wilcoxon rank-sum test, *P* = 9.65 × 10^−3^; Fig. 4f). Human-related ARGs were about four times as abundant in the downstream samples as the river-specific ARGs, clearly indicating the major source of ARGs in the downstream regions. Notably, the river-specific ARGs also increased rather significantly, supporting our previous observation that fecal contamination is not the sole cause of ARG bloom in the downstream regions.

When the bacterial genome database was searched for human-related ARGs, these ARGs were more frequently found in the MGE context (Fig. 4g) and showed broader host ranges at various taxonomic levels (Fig. 4h) compared to river-specific ARGs. These results suggest that mobility and horizontal gene transfer of human-related ARGs between taxa play an important role in the ARG bloom in the downstream regions.

### Phylogenetic distribution of ARGs

**Fig. 5:**
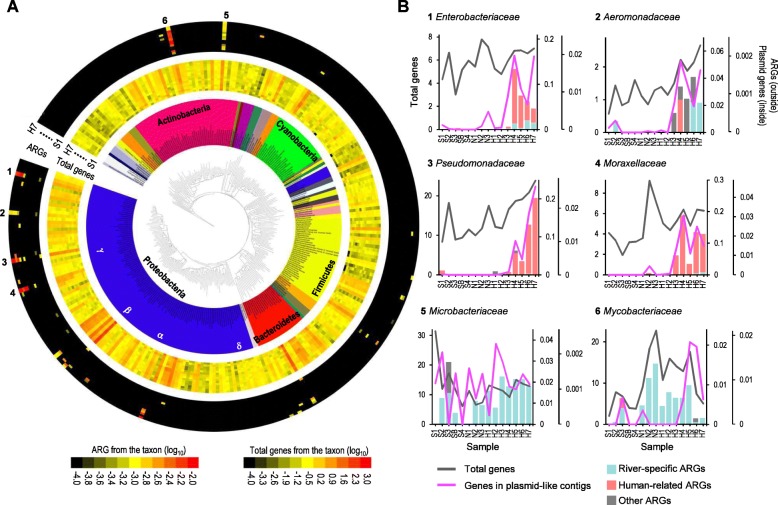
Phylogenetic distribution of ARGs based on taxonomy of contigs carrying ARGs. **a** The phylogenetic distribution and abundances of ARGs (outer heatmaps) and total genes (inner heatmaps) across bacterial families based on the taxonomic classification of metagenome contigs from the samples taken in May 2016. The phylogenetic tree was reconstructed using the maximum-likelihood method based on 16S rRNA gene sequences representative of bacterial families. Heatmaps were generated using the Interactive Tree of Life online tool (http://itol.embl.de). Each circular track in the heatmap represents a sample. **b** The abundance profiles of human-related ARGs, river-specific ARGs, total genes, and genes in plasmid-like contigs in the selected bacterial taxa (marked as 1–6 in Fig. 5a).

Metagenome assembly provided a snapshot of taxonomic distribution of ARGs among the members of the Han River microbial communities. ARGs were detected in limited members of the bacterial phylogeny found among the metagenome contigs (Fig. 5a). A majority of ARGs were found on contigs assigned to Actinobacteria, Bacteroidetes, Firmicutes and Proteobacteria. Six bacterial families were the dominant hosts of river ARGs: *Aeromonadaceae*, *Enterobacteriaceae*, *Moraxellaceae*, and *Pseudomonadaceae*, belonging to Proteobacteria, and *Microbacteriaceae* and *Mycobacteriaceae*, belonging to Actinobacteria. In the families of Proteobacteria, the downstream samples were dramatically enriched for ARGs and plasmid-like contigs compared to levels in the total gene pool (Fig. 5b). In contrast, in the families of Actinobacteria, the abundances of ARGs followed the same patterns observed for total genes (Fig. 5b). ARGs from the contigs assigned to *Enterobacteriaceae*, *Moraxellaceae*, and *Pseudomonadaceae* were mostly human-related, while ARGs from *Microbacteriaceae* and *Mycobacteriaceae* were almost exclusively river-specific (Fig. 5b). These results suggest that the ARG bloom in the downstream regions is mainly driven by human-related ARGs carried by certain proteobacterial members.

### Phenotypic characteristics of ARB isolated in Han River

**Fig. 6:**
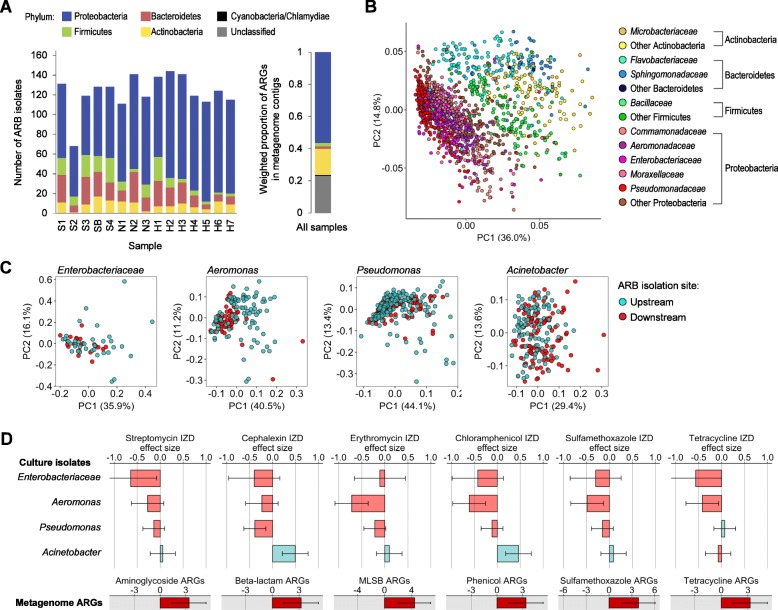
Phylogeny and resistance phenotypes of ARB isolated from the Han River. **a** Phylum-level composition of ARB isolates collected from each sample. The plot on the right shows the relative proportions of bacterial phyla among the metagenome contigs carrying ARGs. **b** Principal component analysis (PCA) plot of resistance phenotypes of ARB. PCA was performed for all ARB isolates based on the Euclidean distances in phenotypic profiles composed of inhibition zone diameters determined by disc diffusion assay. **c** PCA plots based on the resistance phenotypes of upstream and downstream isolates of *Enterobacteriaceae*, *Aeromonas*, *Pseudomonas*, and *Acinetobacter*. **d** Effect sizes for the differences in inhibition zone diameter (IZD) between upstream and downstream isolates of *Enterobacteriaceae*, *Aeromonas*, *Pseudomonas*, and *Acinetobacter*. The bottom plots display the effect sizes for the metagenome-wide ARG densities against the corresponding antibiotic classes. Negative effect sizes indicate decreased IZD of downstream isolates compared to upstream ones.

We constructed a 16S rRNA gene-based phylogeny and analyzed the resistance phenotypes of 1557 ARB strains isolated from the Han River in May of 2016. The phylogenetic composition of these isolates was consistent with the bacterial phyla identified as the major hosts of ARGs based on metagenomics analysis (Fig. 6a). The resistance phenotypes of ARB were strongly clustered by phylum rather than by the geographic location (ANOSIM, *R* = 0.631 and *P* = 0.0002 for phylum, *R* = − 0.022 and *P* = 0.99 for location; Fig. 6b). We compared resistance phenotypes between upstream and downstream isolates of ARB genera (≥ 8 isolates from upstream and downstream, respectively). In the case of the family *Enterobacteriaceae*, several genera, including *Escherichia*, *Klebsiella*, *Enterobacter*, *Citrobacter*, and *Serratia*, were pooled together due to the low numbers of isolates. Among the ten genera and one family evaluated, *Enterobacteriaceae*, *Aeromonas*, *Pseudomonas*, and *Acinetobacter* exhibited significantly different resistance profiles between upstream and downstream isolates (permutational analysis of variance (PERMANOVA), *P* < 0.05; Fig. 6c). These taxa exactly corresponded to the γ-proteobacterial families identified in the metagenomics analysis as major members displaying increases in ARG prevalence downstream (Fig. 5b). Furthermore, we compared the susceptibility of upstream and downstream isolates within each genus or family against six representative antibiotics displaying the most dramatic increases in ARGs (Figs. 1d and 6d). Most of these downstream isolates, except for *Acinetobacter* strains, showed decreased susceptibility (negative effect size) compared to the upstream isolates (Fig. 6d).

## Discussion

Several studies have reported a positive correlation between the abundance of ARGs in the environment and anthropogenic activities [27–29]. In principle, the effect of anthropogenic activities on the environmental resistome could be mediated by two types of processes: input of human-related ARGs into the environment and selection pressure for the carriage of ARGs [30]. The latter process is often hypothesized to promote the spread of mobile ARGs among bacterial communities in the environment. Evaluation of these ecological and evolutionary scenarios in environmental settings has been difficult due to the absence of appropriate data supporting these hypotheses. In the present study, we employed a river model ecosystem exhibiting a resistome succession driven by a gradient of anthropogenic activities at highly populated downstream regions, and we evaluated dynamics in human-related ARGs that occur over the course of such a transition in the river ecosystem.

A recent study based on metagenomics analysis of a human fecal indicator bacteriophage showed that quantitative dynamics of ARGs from anthropogenically impacted environmental samples could be primarily explained by human fecal pollution, implying that input events rather than on-site selection pressures play a critical role in anthropogenic effects on the environmental resistome [19]. Other studies have shown that increases in ARGs in anthropogenically impacted rivers are accompanied by concomitant increases in pathogenic bacteria and human gut microbiome-associated sequences [18]. The present study also showed that both fecal phage and representative fecal bacteria increased in the downstream regions, although these fecal factors were not enough to fully explain the ARG bloom in the downstream regions.

Accordingly, this raises the question of how much of the increase in ARGs in anthropogenically polluted environments is contributed by ARGs introduced from human-related bacteria and how much is contributed by ARGs indigenous to the environment. In the river system studied here, a large proportion of ARGs was shared with the human gut or pathogen resistomes. These human-related ARGs increased more steeply in the downstream regions than the other ARGs found in the river metagenomes, highlighting the fact that these genes are the major components of anthropogenically driven bloom of the river resistome. Notably, river-specific ARGs also increased 4.6-fold in the downstream regions, similar to human-related SCGs, suggesting that fecal input is not the major reason for the ARG bloom.

The association of ARGs with MGEs is known to facilitate the spread of ARGs within and between environments through HGT [30]. Therefore, the localization of ARGs on MGEs has a critical influence on the fate of ARGs in the environment [24]. Many studies have reported elevations in MGE abundance in environments with anthropogenic influences [19, 29]. Class 1 integrons are the most well-established indicator of such an influence [24, 27]. However, the hypothesis that the spread of mobile ARGs is especially relevant to anthropogenically influenced environments has not been systematically examined. In the present study, we observed an increase in MGE abundance and the frequent genetic linkage of ARGs and MGEs in downstream regions. Furthermore, our results suggest that mobile ARGs play a dominant role in the anthropogenic transition of the river resistome. ARG sequences shared among river, human gut, and pathogens were frequently found in MGE contexts and were observed across a broader phylogeny of bacterial genomes. These human-related ARGs were not concomitantly detected with core phylogenetic marker genes. Our results suggest that ARGs rather than ARB are selected and these ARGs are mobilized and transferred laterally between different taxa in the downstream regions under high anthropogenic influences.

The identification of bacteria carrying ARGs or displaying resistance phenotypes is critical for monitoring, risk assessment, and management of the environmental resistome. The taxonomy-resolved structure of the environmental resistome has mostly been evaluated in culture-based studies, which are able to assess resistance phenotypes and genetic determinants of isolates [14, 20]. Recently, several studies have demonstrated host-tracking of the environmental resistome based on the taxonomic classification of metagenomic contigs harboring ARGs [31]. Although metagenomics approaches have advantages over culture-dependent approaches in terms of elucidating comprehensive and unbiased resistome profiles, especially for complex environmental communities, they are limited in terms of providing accurate taxonomic information and solid phenotypic evidence. In the present study, we harnessed both culture-dependent and -independent approaches to generate an integrative picture of ARG host ranges and the phenotype-level resistome. Using a metagenomics approach, we found that the overall host range of the river resistome was limited to a small number of branches across the bacterial phylogeny. Four proteobacterial families were the major hosts of ARGs, and their contigs showed a higher ARG density in downstream regions. It is noteworthy that all four families playing a major role in the downstream resistome encompass clinically important human pathogens. Analysis of resistance phenotypes in over 1500 ARB isolates complemented the taxonomic prediction of ARG hosts based on metagenomic contigs. A recent study showed that ARG profiles derived from functional metagenomics screening and the resistance phenotypes of coliform isolates from a sewage system were correlated [32]. Likewise, in some bacterial taxa whose ARG contents differed considerably between upstream and downstream regions based on metagenomics analysis, such as *Acinetobacter*, *Aeromonas*, *Enterobacteriaceae*, and *Pseudomonas*, we observed significantly different resistance phenotypes between upstream and downstream isolates. Such differences were not observed among isolates belonging to other taxa. In particular, most of these isolates from downstream regions exhibited decreased susceptibility against various classes of antibiotics. This observation was consistent with the increased human-related ARGs from these four proteobacterial lineage at the downstream regions, suggesting that human-related mobile ARGs are horizontally transferred to the environmental relatives of their original hosts and proliferate in the environment. Collectively, our results from both metagenomics-based analysis of ARGs and phenotypic analysis of ARB isolates showed similar trends, validating our robust characterization of river resistome dynamics driven by anthropogenic activities. Addressing what evolutionary mechanisms at the individual genome and pan-genome levels lead to the spread of these particular mobile ARGs and how the environmental resistome in turn influences the resistome in clinical settings are the next steps for better understanding the global dissemination of antibiotic resistance.

## Conclusions

In this study, we evaluated a river model ecosystem exhibiting characteristic resistome dynamics driven by anthropogenic impacts. Snapshots taken from the river continuum under a gradient of anthropogenic pressures provided novel insights into how human activities shape the environmental resistome. Our results demonstrate that fecal contamination could be responsible for the introduction of ARGs into the anthropogenically impacted river resistome, but human-related mobile resistance genes rather than resistant fecal bacteria proliferate in environmental relatives of clinically important proteobacteria.

## Methods

### Sampling and physicochemical measurements

Samples were collected from 15 sites along the Han River over three different seasons in May 2016, August 2016, and February 2017. Each sampling trip was completed within two days, and rainfall was avoided for at least 3 days prior to each sampling. At each of the 15 sampling sites, samples were collected from three spots located within 50 m and mixed to give a total volume of 4–10 L per site. At each spot, river water was collected from the surface to a depth of around 1 m. Water samples were immediately transported to the laboratory and used for further analyses. Temperature, pH, and dissolved oxygen were measured at each sampling site using a ProPlus Multiparameter Instrument (YSI, Yellow Springs, OH, USA). Total phosphate, total nitrogen, and total suspended solid concentrations were analyzed at the Korea Environment and Water Works Institute (Seoul, Korea). Details on sample metadata are provided in Additional file 1: Table S1.

### Environmental DNA extraction

Each water sample was pre-filtered through a 10-μm pore nylon membrane (Millipore, Billerica, MA, USA) to remove large particles and then filtered through a 0.2-μm pore size mixed cellulose ester membrane (Advantec, Tokyo, Japan). The membranes were stored at − 80 °C for DNA extraction. Environmental DNA was extracted from the membranes using DNeasy PowerWater kit (Qiagen, Hilden, Germany) according to the manufacturer’s instructions. The same DNA samples were used for 16S rRNA gene amplicon and metagenome sequencing and HT-qPCR.

### Shotgun metagenome and 16S rRNA gene amplicon sequencing

Shotgun libraries were prepared using the Illumina TruSeq DNA PCR-free library preparation kit (San Diego, CA, USA) according to the manufacturer’s instructions. Amplicon libraries targeting the V3-V4 region of the bacterial 16S rRNA gene were prepared using the primers 341F (5′-CCT ACG GGN GGC WGC AG-3′) and 805R (5′-GAC TAC HVG GGT ATC TAA TCC-3′). Shotgun and 16S rRNA gene amplicon libraries were sequenced by 151-bp paired-end sequencing on a HiSeq 4000 platform and 300-bp paired-end sequencing on a MiSeq platform, respectively, at Macrogen (Seoul, Korea). Accession numbers for sequencing data are provided in Additional file 2: Table S3.

### High-throughput quantitative PCR

HT-qPCR with 343 ARG-targeted and 36 MGE-targeted primer sets [28] was conducted using the SmartChip Real-time PCR system (Takara, Shiga, Japan). PCR reactions, C_T_ calculations, and analysis of the relative abundances ARGs and MGEs were performed as previously described [28, 29]. All reactions were performed in triplicate.

### Analysis of bacterial 16S rRNA gene amplicon data

Paired-end reads from 16S rRNA gene amplicons were merged and clustered into OTUs using a 97% sequence identify cut-off with UCLUST [33]. Taxonomic classification of OTUs was performed using the RDP classifier based on Ribosomal Database Project-II (http://rdp.cme.msu.edu) as a reference. Chao 1 and phylogenetic diversity indices were estimated from OTU tables after normalization to 36,428 reads per sample, which was the lowest number of reads per sample. All computations were performed using the QIIME pipeline (https://qiime2.org/) [34].

### Databases for ARGs and MGEs

The Comprehensive Antibiotic Resistance Database (CARD) [35] was downloaded in October 2017 and modified for metagenomics analysis. Briefly, among the 2177 protein homolog models, we removed 18 models known as global regulators. The remaining 2159 reference proteins were sorted into 751 non-redundant ARG names based on the clustering of sequences at an 80% global identity, phylogenetic relationships within homologous clusters, and gene annotations. These non-redundant ARG names were used for the analysis of ARG profiles. The list and classification of ARGs in our modified CARD are provided in Additional file 3: Table S4. Integron integrase sequences were collected from the NCBI protein database using the following search terms: “IntI”, “integron integrase”, and “IntI*” and a filter selective for bacterial RefSeq records. Integrase sequences were clustered at 100% identity and classified into classes 1–4 based on a phylogenetic tree reconstructed with XerC (NP_418256.1) and XerD (NP_417370.1) sequences. Transposase sequences were collected in the same way using “transposase” as a search term. Out of 273,150 collected sequences, 56,821 transposases associated with insertion sequence (IS) elements based on feature descriptions were used as the reference IS transposase database.

### Metagenome assembly, annotation, and gene profiling

Shotgun sequencing reads were pre-processed by removing adapter sequences and filtering out low-quality reads using FaQCs [36]. Metagenome assembly was performed for each sample using IDBA-UD v1.1 [37]. After assembly, reads were mapped to the contigs by BWA-MEM [38], and the average coverage depth of each contig was calculated using SAMtools v0.1.19 [39]. Taxonomic classification of metagenome contigs was performed using Kraken v1.0 [40] and the reference database containing complete genomes of bacteria, archaea, viruses, fungi, and other eukaryotic microbes downloaded from the NCBI in October 2017. Protein-coding sequences (CDSs) in contigs were identified using Prodigal v2.6 [41] with the “-p meta” option. Predicted CDSs were annotated based on the COG [42], CARD [35], and the MGE database constructed in this study using blastp as implemented in DIAMOND v0.9.19 [43]. For annotation of ARGs, cut-offs of 80% identity and 80% query coverage were applied. Cut-offs of 80% identity and an amino acid length of 25 for MGE annotation and an *E*-value of 1E-7 for COG annotation were employed. The normalized abundance of each CDS was calculated as the coverage depth of the contig containing each CDS divided by the average read depth of 40 universal SCGs [44]. Plasmid-like contigs were identified by blastn search against 8323 plasmid sequences downloaded from the NCBI RefSeq database. Metagenome contigs that had hits with ≥ 90% identity and an alignment length ≥ 1000 bp in the plasmid database were identified as plasmid-like contigs. For profiling of ARGs from unassembled metagenome reads, we aligned the reads to 2159 reference ARG sequences using blastx as implemented in DIAMOND v0.9.19 [43]. Blastx hits were filtered using cut-offs of 90% identity and an amino acid length of 25. Reads per kilobase of sequence per million mapped reads (RPKM) were calculated for each reference sequence and normalized by the average RPKM of 40 SCGs.

### Analyses of data from public metagenomes and genomes

Human gut metagenome data from healthy adult subjects from various countries were selected based on the previously published meta-analysis study [25] and downloaded from NCBI Sequence Read Archive (SRA). Human gut samples analyzed in this study included 10 Australian (PRJEB6092), 10 Chinese (PRJEB5224 and SRP008047), 10 Danish (PRJEB2054), 9 Spanish (PRJEB2054), 10 Swedish (PRJEB1786), and 36 Korean (PRJEB1690) individuals. For each sample, we downloaded the SRA file, extracted the fastq file using SRA toolkit v2.9 (https://github.com/ncbi/sra-tools), and assembled contigs using MEGAHIT v1.1.3 [45]. A list of publicly available genomes of human pathogenic bacteria was obtained from the PATRIC database [26] as of 16 April 2018. We selected genomes with “WGS” or “complete” status and manually inspected the metadata table downloaded from the PATRIC database to identify reliable sets of human pathogen genomes. If a bacterial species was isolated from blood or other body fluids, or there was a comment about clinical symptoms, the species was tagged as a “true” pathogen. Genomes of species not tagged as a “true” pathogen were excluded. Draft genomes with >100 contigs were also excluded. For the remaining 24,428 genomes, contig sequences were downloaded from the NCBI nucleotide database. The bacterial genome dataset including non-pathogenic bacteria was obtained for the whole set of 97,235 bacterial genomes listed in the UniProt Proteomes [46] as of December 2018. Genomes were annotated for ARGs, COGs, and MGEs using the methods described for the annotation of metagenomic contigs. Contigs of public genomes were identified as plasmid-like contigs when ≥ 90% of the contig length aligned with ≥ 90% identity to the plasmid reference database by blastn. An OTU table released by the Earth Microbiome Project [21] based on quality-filtered reads longer than 150-bp and containing 12,536 samples was downloaded from the project’s FTP site (ftp://ftp.microbio.me/emp/release1/otu_tables/). Representative human fecal OTUs were selected according to their median abundances in human distal gut samples. Twenty-seven top ranked OTUs comprising > 50% of total read numbers from human distal gut samples were defined as representative human fecal OTUs (Additional file 1: Table S2).

Nucleotide sequences of ARGs annotated in metagenomic and genomic contigs were pooled together for clustering analysis. Partial ARG sequences with < 90% coverage by blastx search against CARD were removed. The remaining sequences were clustered with a 99% identity cut-off using CD-HIT-est v4.6 [47]. Likewise, clustering at a 99% identity cut-off was performed for each SCG. The resulting clusters were used to construct HGT networks of ARGs and identify shared ARGs and SCGs between the river, human gut, and pathogen resistomes.

### Isolation, identification, and phenotypic profiling of ARB

ARB were isolated from the samples collected in May 2016 using Mueller Hinton agar supplemented with various antibiotics. Concentrations of antibiotics in the selective media were one- or twofold higher than the clinical breakpoints suggested by the Clinical and Laboratory Standards Institute (CLSI) guidelines 2011: gentamicin (8 mg/L), amoxicillin (16 mg/L), cephalexin (32 mg/L), tetracycline (4 mg/L), erythromycin (2 mg/L), chloramphenicol (16 mg/L), ciprofloxacin (2 mg/L), lincomycin (2 mg/L), vancomycin (4 mg/L), and sulfamethoxazole (40 mg/L). Strains were identified based on their 16S rRNA gene sequences using the EzBioCloud database (https://www.ezbiocloud.net/resources/16s_download) [48]. Susceptibility against 18 different antibiotics (Liofilchem, Roseto, Italy) was tested by the disc diffusion assay according to the CLSI guidelines 2011. The amount of antibiotic in each disc is as follows (in μg): gentamicin 10, streptomycin 10, amoxicillin 10, cephalexin 30, meropenem 10, tetracycline 30, erythromycin 15, tylosin 30, chloramphenicol 30, ciprofloxacin 5, clindamycin 2, vancomycin 30, sulfamethoxazole 50, trimethoprim 5, linezolid 10, rifampicin 5, colistin 10, and fosfomycin 200.

### Statistical analyses

Correlations between ARG density and other sample parameters were evaluated by Pearson’s and Spearman’s correlation tests. The significance of differences in the compositions of ARGs, OTUs, and COGs within each of the geographic and seasonal sample categories was tested using analysis of similarities (ANOSIM) as implemented in the *vegan* R package [49]. The significance of differences in the overall resistance phenotypes of ARB isolates according to geographic location or taxonomic affiliation was tested using ANOSIM and permutational multivariate analysis of variance (PERMANOVA) as implemented in the *vegan* R package [49]. Differences in susceptibility to each individual antibiotic between upstream and downstream isolates were assessed using the *t* test in R [50].

## Supplementary information

**Additional file 1: **Supplementary information file. **Figures S1.** Correlations of ARG density with microbiological, genetic, physicochemical, geographic, and socio-economic parameters. **Figure S2.** Proportions of losses and gains of ARGs and OTUs between river samples according to geographic distance. **Figure S3.** Relative abundances of mobile genetic elements (MGEs) in the metagenome contigs of Han River. **Figure S4.** Co-occurrence of ARGs and MGEs in metagenome contigs. **Figure S5.** Horizontal gene transfer networks of ARGs inferred from pathogen genomes and river metagenomes. **Figure S6.** Comparison of ARG compositions between river and human gut metagenomes. **Table S1.** Sample metadata. **Table S2.** Representative fecal OTUs selected from the OTU table of the Earth Microbiome Project.

**Additional file 2: Table S3.** Sequencing data statistics and accessions.

**Additional file 3: Table S4.** Modification of CARD.

## Data Availability

The raw sequence data from metagenome shotgun and 16S rRNA gene amplicon sequencing were submitted to NCBI SRA under BioProject accession number PRJNA530373 (https://www.ncbi.nlm.nih.gov/bioproject/530373).

## Abbreviations

ANOSIM: Analysis of similarities
ARB: Antibiotic-resistant bacteria
ARG: Antibiotic resistance gene
CARD: Comprehensive Antibiotic Resistance Database
COGs: Clusters of orthologous genes
HGT: Horizontal gene transfer
MGE: Mobile genetic element
HT-qPCR: High-throughput quantitative PCR
MLSB: Macrolide-Lincosamide-Streptogramin B
OTU: Operational taxonomic unit
PATRIC: Pathosystems Resource Integration Center
PERMANOVA: Permutational analysis of variance
RPKM: Reads per kilobase of sequence per million mapped reads
SCG: Single-copy gene
SRA: Sequence Read Archive

## References

[1] Abubakar I, Zignol M, Falzon D, Raviglione M, Ditiu L, Masham S, Adetifa I, Ford N, Cox H, Lawn SD (2013). Drug-resistant tuberculosis: time for visionary political leadership. Lancet Infect Dis..

[2] Cantón R, Akóva M, Carmeli Y, Giske CG, Glupczynski Y, Gniadkowski M, Livermore DM, Miriagou V, Naas T, Rossolini GM (2012). Rapid evolution and spread of carbapenemases among *Enterobacteriaceae* in Europe. Clin Microbiol Infect..

[3] Gygli SM, Borrell S, Trauner A, Gagneux S (2017). Antimicrobial resistance in *Mycobacterium tuberculosis*: mechanistic and evolutionary perspectives. FEMS Microbiol Rev..

[4] Navon-Venezia S, Kondratyeva K, Carattoli A (2017). *Klebsiella pneumoniae*: a major worldwide source and shuttle for antibiotic resistance. FEMS Microbiol Rev..

[5] McCarthy AJ, Loeffler A, Witney AA, Gould KA, Lloyd DH, Lindsay JA (2014). Extensive horizontal gene transfer during *Staphylococcus aureus* co-colonization in vivo. Genome Biol Evol..

[6] Caro-Quintero A, Konstantinidis KT (2014). Inter-phylum HGT has shaped the metabolism of many mesophilic and anaerobic bacteria. ISME J..

[7] Hehemann J-H, Correc G, Barbeyron T, Helbert W, Czjzek M, Michel G (2010). Transfer of carbohydrate-active enzymes from marine bacteria to Japanese gut microbiota. Nature..

[8] Jiang X, Ellabaan MMH, Charusanti P, Munck C, Blin K, Tong Y, Weber T, Sommer MOA, Lee SY (2017). Dissemination of antibiotic resistance genes from antibiotic producers to pathogens. Nat Commun..

[9] Forsberg KJ, Reyes A, Wang B, Selleck EM, Sommer MOA, Dantas G (2012). The shared antibiotic resistome of soil bacteria and human pathogens. Science..

[10] Nordmann P, Poirel L (2005). Emergence of plasmid-mediated resistance to quinolones in *Enterobacteriaceae*. J Antimicrob Chemother..

[11] Canton R, Gonzalez-Alba JM, Galán JC. CTX-M enzymes: origin and diffusion. Front Microbiol. 2012;3(110). 10.3389/fmicb.2012.00110 PMC3316993 22485109

[12] Wang R, van Dorp L, Shaw LP, Bradley P, Wang Q, Wang X, Jin L, Zhang Q, Liu Y, Rieux A (2018). The global distribution and spread of the mobilized colistin resistance gene *mcr*-*1*. Nat Commun..

[13] McEwen SA, Collignon PJ. Antimicrobial resistance: a one health perspective. Microbiol Spectr. 2018;6(2). 10.1128/microbiolspec.arba-0009-2017 PMC11633550 29600770

[14] D'Costa VM, McGrann KM, Hughes DW, Wright GD (2006). Sampling the antibiotic resistome. Science..

[15] Pal C, Bengtsson-Palme J, Kristiansson E, Larsson DGJ (2016). The structure and diversity of human, animal and environmental resistomes. Microbiome..

[16] Forsberg KJ, Patel S, Gibson MK, Lauber CL, Knight R, Fierer N, Dantas G (2014). Bacterial phylogeny structures soil resistomes across habitats. Nature..

[17] Savio D, Sinclair L, Ijaz UZ, Parajka J, Reischer GH, Stadler P, Blaschke AP, Blöschl G, Mach RL, Kirschner AKT (2015). Bacterial diversity along a 2600 km river continuum. Environ Microbiol..

[18] Zhang S-Y, Tsementzi D, Hatt JK, Bivins A, Khelurkar N, Brown J, Tripathi SN, Konstantinidis KT (2019). Intensive allochthonous inputs along the Ganges River and their effect on microbial community composition and dynamics. Environ Microbiol..

[19] Karkman A, Pärnänen K, Larsson DGJ (2019). Fecal pollution can explain antibiotic resistance gene abundances in anthropogenically impacted environments. Nat Commun..

[20] Amos GCA, Ploumakis S, Zhang L, Hawkey PM, Gaze WH, Wellington EMH (2018). The widespread dissemination of integrons throughout bacterial communities in a riverine system. ISME J..

[21] Thompson LR, Sanders JG, McDonald D, Amir A, Ladau J, Locey KJ, Prill RJ, Tripathi A, Gibbons SM, Ackermann G (2017). A communal catalogue reveals Earth’s multiscale microbial diversity. Nature..

[22] Brito IL, Yilmaz S, Huang K, Xu L, Jupiter SD, Jenkins AP, Naisilisili W, Tamminen M, Smillie CS, Wortman JR (2016). Mobile genes in the human microbiome are structured from global to individual scales. Nature..

[23] Kintses B, Méhi O, Ari E, Számel M, Györkei Á, Jangir PK, Nagy I, Pál F, Fekete G, Tengölics R (2019). Phylogenetic barriers to horizontal transfer of antimicrobial peptide resistance genes in the human gut microbiota. Nat Microbiol..

[24] Gillings MR, Gaze WH, Pruden A, Smalla K, Tiedje JM, Zhu Y-G (2014). Using the class 1 integron-integrase gene as a proxy for anthropogenic pollution. ISME J..

[25] Mancabelli L, Milani C, Lugli GA, Turroni F, Ferrario C, van Sinderen D, Ventura M (2017). Meta-analysis of the human gut microbiome from urbanized and pre-agricultural populations. Environ Microbiol..

[26] Wattam AR, Abraham D, Dalay O, Disz TL, Driscoll T, Gabbard JL, Gillespie JJ, Gough R, Hix D, Kenyon R (2014). PATRIC, the bacterial bioinformatics database and analysis resource. Nucleic Acids Res..

[27] Gaze WH, Zhang L, Abdouslam NA, Hawkey PM, Calvo-Bado L, Royle J, Brown H, Davis S, Kay P, Boxall ABA (2011). Impacts of anthropogenic activity on the ecology of class 1 integrons and integron-associated genes in the environment. ISME J..

[28] Wang F-H, Qiao M, Su J-Q, Chen Z, Zhou X, Zhu Y-G (2014). High throughput profiling of antibiotic resistance genes in urban park soils with reclaimed water irrigation. Environ Sci Technol..

[29] Zhu Y-G, Zhao Y, Li B, Huang C-L, Zhang S-Y, Yu S, Chen Y-S, Zhang T, Gillings MR, Su J-Q (2017). Continental-scale pollution of estuaries with antibiotic resistance genes. Nat Microbiol..

[30] Larsson DGJ, Bengtsson-Palme J, Kristiansson E. Environmental factors influencing the development and spread of antibiotic resistance. FEMS Microbiol Rev. 2017;42(1). 10.1093/femsre/fux053 PMC5812547 29069382

[31] Chu BTT, Petrovich ML, Chaudhary A, Wright D, Murphy B, Wells G, Poretsky R (2018). Metagenomics reveals the impact of wastewater treatment plants on the dispersal of microorganisms and genes in aquatic sediments. Appl Environ Microbiol..

[32] Li A-D, Ma L, Jiang X-T, Zhang T (2017). Cultivation-dependent and high-throughput sequencing approaches studying the co-occurrence of antibiotic resistance genes in municipal sewage system. Appl Microbiol Biotechnol..

[33] Edgar RC (2010). Search and clustering orders of magnitude faster than BLAST. Bioinformatics..

[34] Caporaso JG, Kuczynski J, Stombaugh J, Bittinger K, Bushman FD, Costello EK, Fierer N, Peña AG, Goodrich JK, Gordon JI (2010). QIIME allows analysis of high-throughput community sequencing data. Nat Methods..

[35] Jia B, Raphenya AR, Alcock B, Waglechner N, Guo P, Tsang KK, Lago BA, Dave BM, Pereira S, Sharma AN (2017). CARD 2017: expansion and model-centric curation of the comprehensive antibiotic resistance database. Nucleic Acids Res..

[36] Lo C-C, Chain PSG (2014). Rapid evaluation and quality control of next generation sequencing data with FaQCs. BMC Bioinformatics..

[37] Peng Y, Leung HCM, Yiu SM, Chin FYL (2012). IDBA-UD: a de novo assembler for single-cell and metagenomic sequencing data with highly uneven depth. Bioinformatics..

[38] Li H, Durbin R (2010). Fast and accurate long-read alignment with Burrows–Wheeler transform. Bioinformatics..

[39] Li H, Handsaker B, Wysoker A, Fennell T, Ruan J, Homer N, Marth G, Abecasis G, Durbin R (2009). 1000 Genome Project Data Processing Subgroup. The sequence alignment/map format and SAMtools. Bioinformatics..

[40] Wood DE, Salzberg SL (2014). Kraken: ultrafast metagenomic sequence classification using exact alignments. Genome Biol..

[41] Hyatt D, Chen G-L, LoCascio PF, Land ML, Larimer FW, Hauser LJ (2010). Prodigal: prokaryotic gene recognition and translation initiation site identification. BMC Bioinformatics..

[42] Tatusov RL, Galperin MY, Natale DA, Koonin EV (2000). The COG database: a tool for genome-scale analysis of protein functions and evolution. Nucleic Acids Res..

[43] Buchfink B, Xie C, Huson DH (2014). Fast and sensitive protein alignment using DIAMOND. Nat Methods..

[44] Mende DR, Sunagawa S, Zeller G, Bork P (2013). Accurate and universal delineation of prokaryotic species. Nat Methods..

[45] Li D, Liu C-M, Luo R, Sadakane K, Lam T-W (2015). MEGAHIT: an ultra-fast single-node solution for large and complex metagenomics assembly via succinct de Bruijn graph. Bioinformatics..

[46] The UniProt Consortium (2018). UniProt: a worldwide hub of protein knowledge. Nucleic Acids Res..

[47] Fu L, Niu B, Zhu Z, Wu S, Li W (2012). CD-HIT: accelerated for clustering the next-generation sequencing data. Bioinformatics..

[48] Yoon S-H, Ha S-M, Kwon S, Lim J, Kim Y, Seo H, Chun J (2017). Introducing EzBioCloud: a taxonomically united database of 16S rRNA gene sequences and whole-genome assemblies. Int J Syst Evol Microbiol..

[49] Oksanen J, Blanchet FG, Friendly M, Kindt R, Legendre P, McGlin D, Minchin PR, O’Hara RB, Simpson GL, Solymos P, et al. Vegan: community ecology package. R package version. 2019;25-4.

[50] Core R. Team. R: A language and environment for statistical computing. Vienna, Austria: R Foundation for Statistical. Computing. 2013.

