# Supplementary material for: Mobile resistome of human gut and pathogen drives anthropogenic bloom of antibiotic resistance
Source: Microbiome. 2020 Jan 7;8:2. doi: 10.1186/s40168-019-0774-7 (PMC6947943; doi:10.1186/s40168-019-0774-7)
Supplement: Supplementary file 2 — Additional file 1: Supplementary information file. Figures S1. Correlations of ARG density with microbiological, genetic, physicochemical, geographic, and socio-economic parameters. Figure S2. Proportions of losses and gains of ARGs and OTUs between river samples according to geographic distance. Figure S3. Relative abundances of mobile genetic elements (MGEs) in the metagenome contigs of Han River. Figure S4. Co-occurrence of ARGs and MGEs in metagenome contigs. Figure S5. Horizontal gene transfer networks of ARGs inferred from pathogen genomes and river metagenomes. Figure S6. Comparison of ARG compositions between river and human gut metagenomes. Table S1. Sample metadata. Table S2. Representative fecal OTUs selected from the OTU table of the Earth Microbiome Project. [file 40168_2019_774_MOESM2_ESM.pdf]

**Additional File 1 for**

# Mobile resistome of human gut and pathogen drives anthropogenic bloom of antibiotic resistance

Kihyun Lee[1†], Dae-Wi Kim[1†], Do-Hoon Lee[1], Yong-Seok Kim[1], Ji-Hye Bu[1], Ju-Hee Cha[1], Cung Nawl Thawng[1], Eun-Mi Hwang[1], Hoon Je Seong[1], Woo Jun Sul[1], Elizabeth M. H. Wellington[2], Christopher Quince[3], Chang-Jun Cha[1*]

*Correspondence: cjcha@cau.ac.kr

**This file includes:**

**Figures S1.** Correlations of ARG density with microbiological, genetic, physicochemical, geographic, and socio-economic parameters.

**Figure S2.** Proportions of losses and gains of ARGs and OTUs between river samples according to geographic distance.

**Figure S3.** Relative abundances of mobile genetic elements (MGEs) in the metagenome contigs of Han River.

**Figure S4.** Co-occurrence of ARGs and MGEs in metagenome contigs.

**Figure S5.** Horizontal gene transfer networks of ARGs inferred from pathogen genomes and river metagenomes.

**Figure S6.** Comparison of ARG compositions between river and human gut metagenomes.

**Table S1.** Sample metadata.

**Table S2.** Representative fecal OTUs selected from the OTU table of the Earth Microbiome Project.

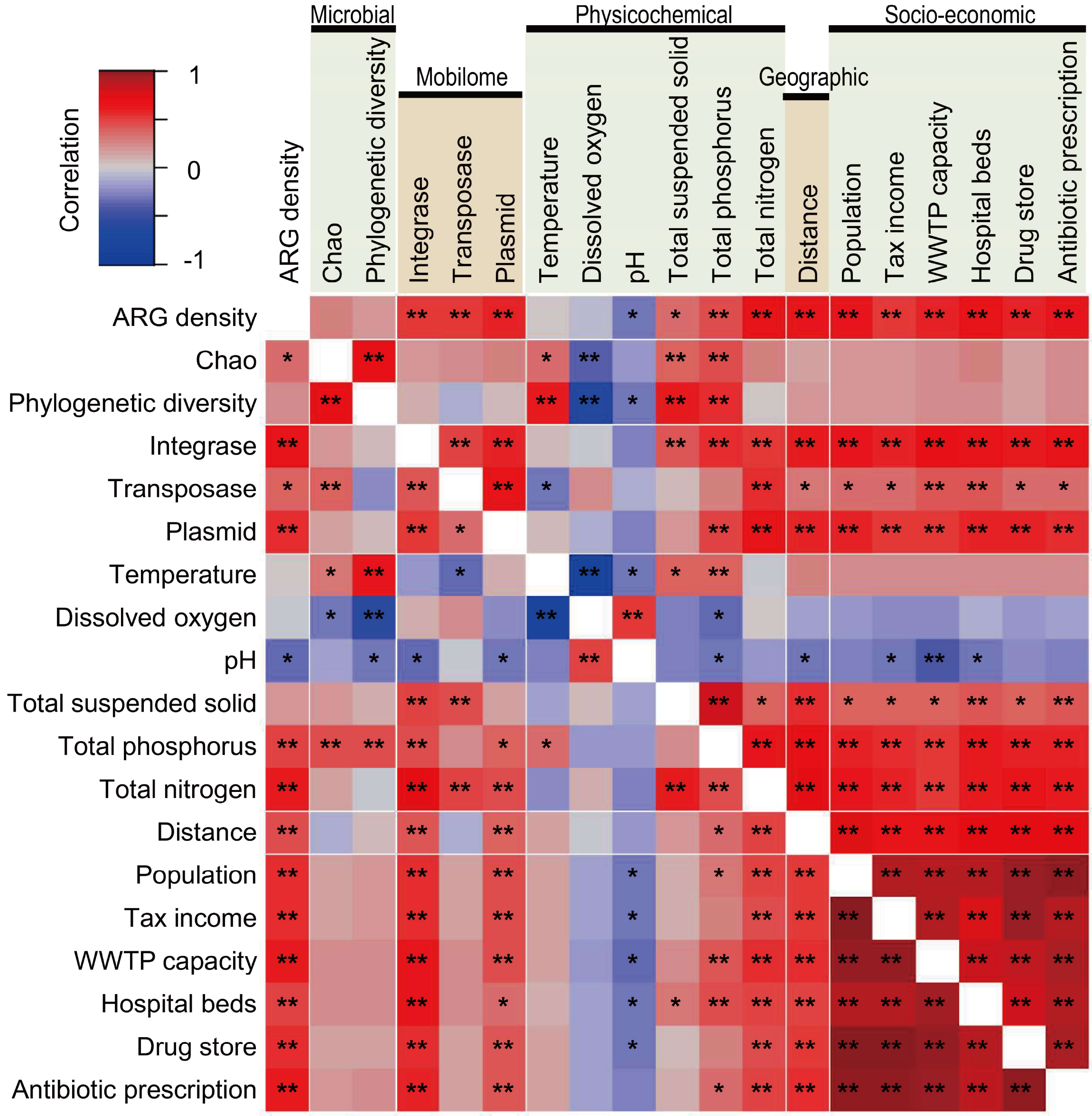


**Figure S1.** Correlations of ARG density with microbiological, genetic, physicochemical, geographic, and socio-economic parameters. Pairwise Pearson's and Spearman's correlations were assessed for various sample parameters. Values measured from 45 samples from three different seasons were used as input for correlation analyses. Pearson's correlation and Spearman's rho values are shown as color gradients in lower and upper triangles, respectively. Significance is indicated by * ($P < 0.05$) and ** ($P < 0.01$). Distance from H7 was scaled from 0 (S1) to 1 (H7). WWTP, wastewater treatment plant.

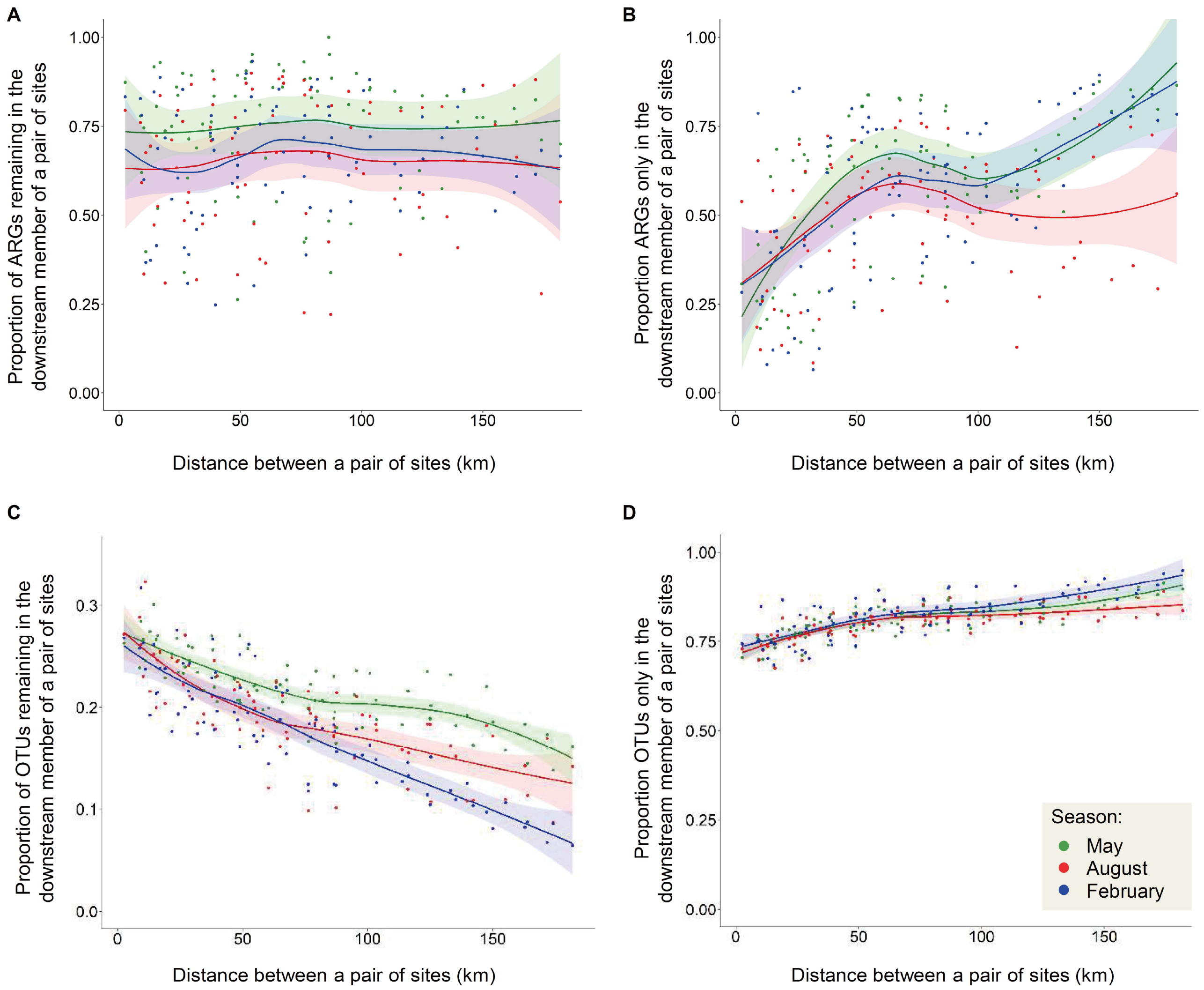


**Figure S2.** Proportions of losses and gains of ARGs and OTUs between river samples according to geographic distance. **a** Distance-decay curve of the proportion of ARGs (clustered at 95% identity) remaining in the downstream member of a pair of sites for comparison. **b** Proportion of ARGs only in the downstream member of a pair of sites for comparison. **c** Distance-decay curve of the proportion of OTUs remaining in the downstream member of a pair of sites for comparison. **d** Proportion of OTUs only in the downstream member of a pair of sites for comparison.

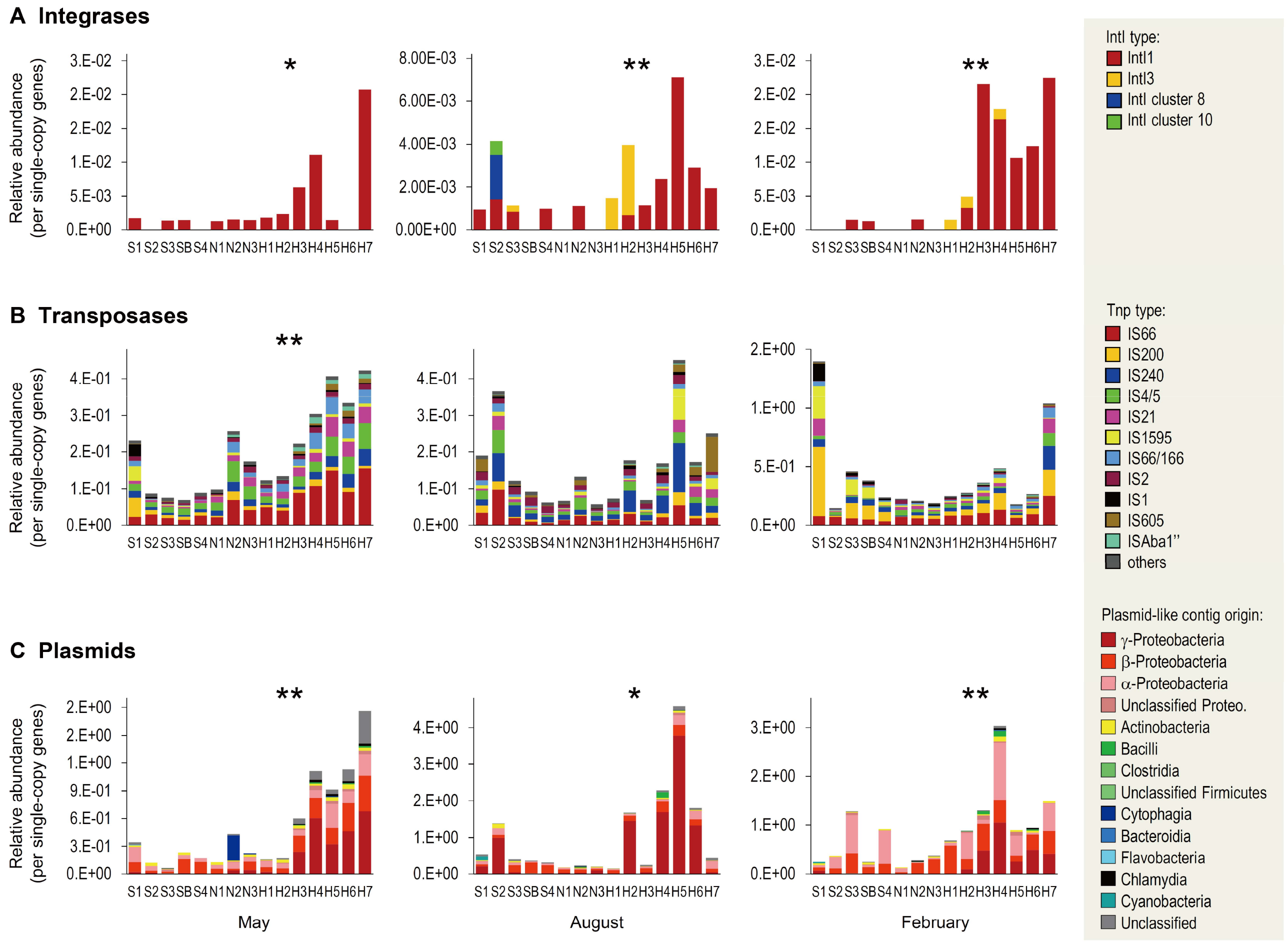


**Figure S3.** Relative abundances of mobile genetic elements (MGEs) in the metagenome contigs of Han River. **a** The relative abundances of integron integrase genes (*intI*) in the metagenome contigs. **b** The relative abundances of transposase genes in the metagenome contigs. **c** The relative abundances of plasmids determined by the abundances of all genes in plasmid-like contigs. For all three MGEs, relative abundance was normalized by the average abundance of universal single-copy genes. The significance level of the difference between upstream and downstream samples is shown above each plot (*$P < 0.05$, **$P < 0.01$, $t$ test). For *intI* data, $t$ test was performed for the abundance of *intI1* rather than the total *intI* abundance.

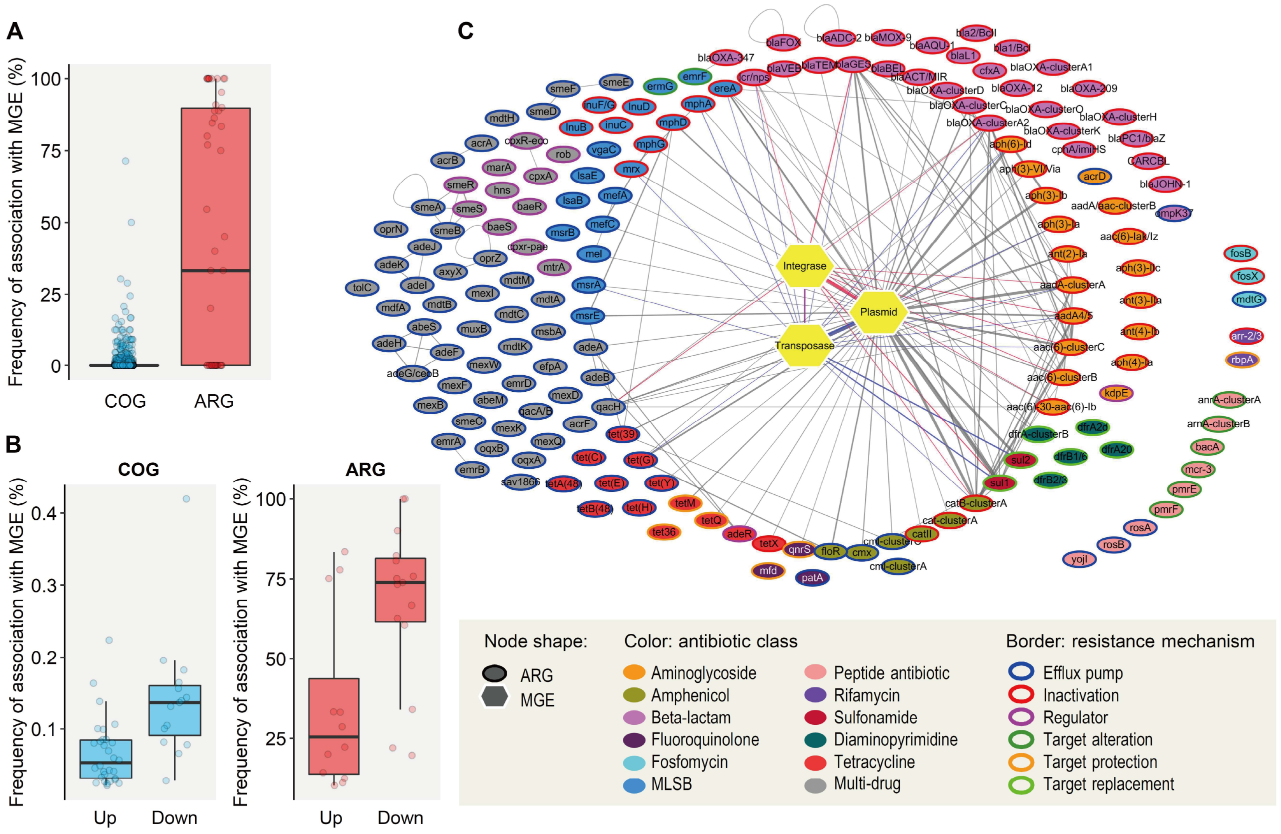


**Figure S4**. Co-occurrence of ARGs and MGEs in metagenome contigs. **a** Boxplot of frequency at which each ARG or COG was detected with MGEs (integrase, transposase, and plasmid) in the same metagenome contig. The frequency was calculated only for gene families (either ARGs or COGs) detected in five or more contigs. **b** Co-occurrence frequencies of ARGs and MGEs (right) and COGs and MGEs (left) in upstream (Up) and downstream (Down) river samples. **c** Network of co-occurrence of ARGs and MGEs on the same contig. Hexagonal nodes represent three types of MGEs. Oval nodes represent ARGs. Edges represent the co-occurrence of two nodes (ARGs or MGEs) in the same contig; the edge width is proportional to the number of cases. Edges involving integrase are in red. Edges involving transposase are in blue. Plasmid edges are in gray.

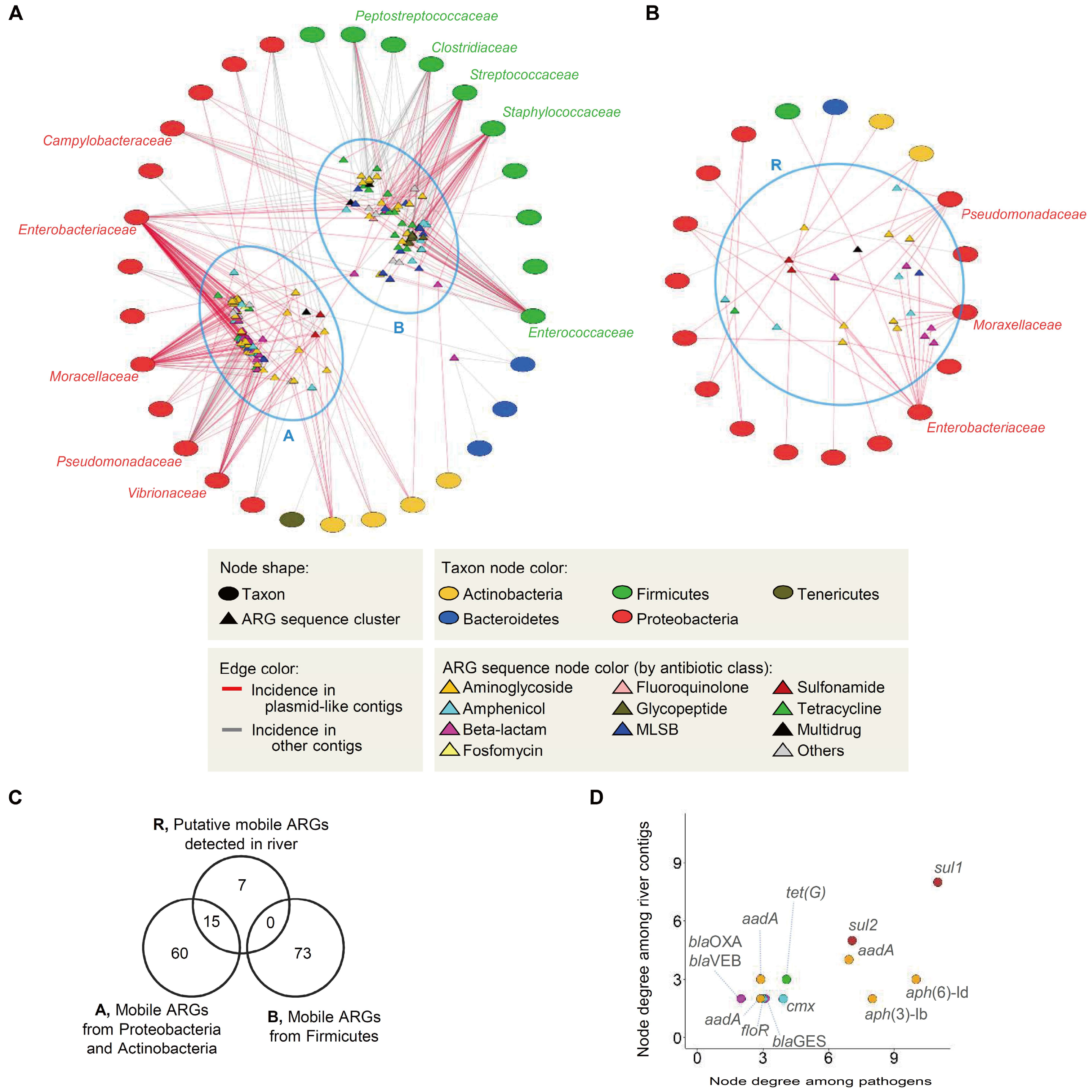


**Figure S5.** Horizontal gene transfer networks of ARGs inferred from pathogen genomes and river metagenomes. **a** Network of bacterial pathogen genomes and ARGs. Taxon nodes represent pathogen genomes grouped at the family level. ARG nodes represent clusters of ARG sequences that share ≥ 99% nucleotide identity. Edges indicate detection of ARGs in the genomes included in the taxon nodes. The occurrence of the same ARG across different taxon nodes was considered evidence of horizontal transfer of ARGs. Two blue circles encompassing each set of ARG nodes were designated as “set A (mobile ARGs from Proteobacteria and

Actinobacteria)" and "set B (mobile ARGs from Firmicutes)". **b** Network of river metagenome contigs and ARGs. Taxon nodes represent metagenome contigs assigned at the family level. Edges indicate detection of ARGs in the metagenome contigs. A blue circle surrounding ARG nodes was designated as "set R (mobile ARGs detected in river metagenomes)". **c** Venn diagram displaying the numbers of ARG nodes shared between the HGT networks of pathogen genomes and river metagenomes. **d** Comparison of node degrees from the HGT networks of pathogen genomes and river metagenomes. Node degree indicates the number of taxon nodes connected to ARGs in each network.

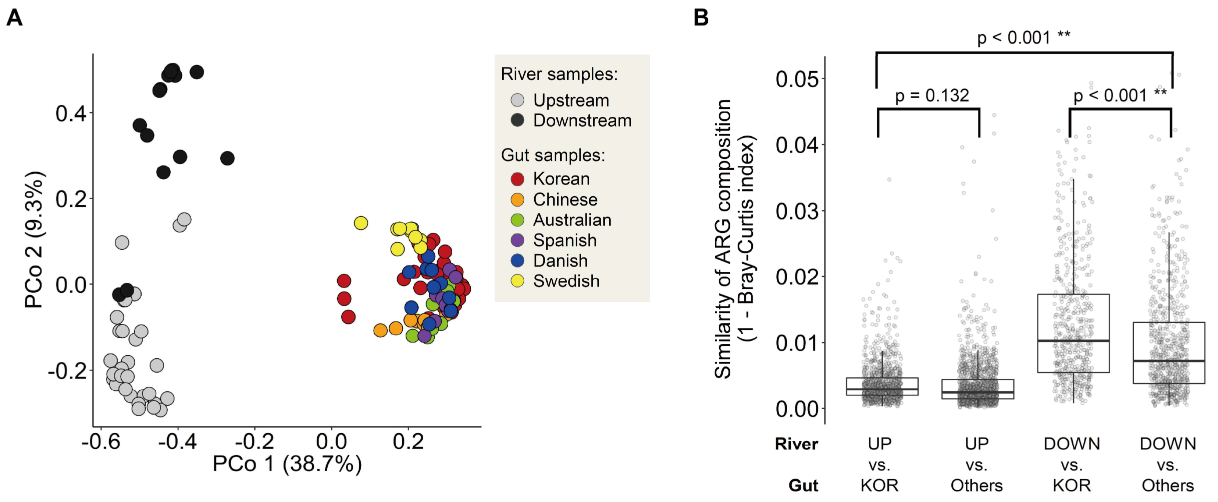


**Figure S6**. Comparison of ARG compositions between river and human gut metagenomes. **a** Principal coordinate analysis of ARG compositions of the Han River and the human gut metagenomes from several countries. **b** Bray–Curtis similarity of ARG compositions between the river and gut metagenomes: upstream river vs. Korean gut, upstream river vs. non-Korean gut, downstream river vs. Korean gut, and downstream river vs. non-Korean gut. Results with P-values less than 0.01 (**) were considered statistically significant.

**Table S1.** Sample metadata.

| Site name | GPS position | Sampling time | Physicochemical parameters | | | | | |
|---|---|---|---|---|---|---|---|---|
| | | | Temperature (°C) | pH | Dissolved oxygen (mg/L) | Total nitrogen (mg/L) | Total phosphate (mg/L) | Total suspended solids (mg/L) |
| S1 | 37°26'0.51" 128°39'27.18" | May 2016 | 18.5 | 7.7 | 9.7 | 1.9 | 0.025 | 4.8 |
| | | Aug. 2016 | 16.5 | 7.9 | 6.4 | 2.3 | 0.086 | 29.0 |
| | | Feb. 2017 | 4.4 | 8.2 | 13.2 | 2.9 | 0.006 | 1.0 |
| S2 | 37°0'22.96 127°59'2.4" | May 2016 | 11.4 | 7.7 | 11.5 | 2.3 | 0.008 | 1.4 |
| | | Aug. 2016 | 18.7 | 7.5 | 3.3 | 2.8 | 0.016 | 1.2 |
| | | Feb. 2017 | 4.8 | 8.3 | 10.7 | 1.9 | 0.006 | 0.7 |
| S3 | 37°17'23.51" 127°39'5.92" | May 2016 | 17.2 | 8.0 | 11.1 | 2.2 | 0.027 | 7.7 |
| | | Aug. 2016 | 20.9 | 8.2 | 9.1 | 2.5 | 0.011 | 1.4 |
| | | Feb. 2017 | 3.3 | 8.3 | 13.8 | 2.5 | 0.026 | 0.9 |
| SB | 37°23'46.06" 127°32'40.09 | May 2016 | 18.2 | 8.0 | 11.9 | 2.4 | 0.045 | 13.5 |
| | | Aug. 2016 | 21.9 | 8.0 | 8.1 | 2.3 | 0.013 | 2.8 |
| | | Feb. 2017 | 3.6 | 8.5 | 12.5 | 2.8 | 0.012 | 1.7 |
| S4 | 37°29'1.61" 127°29'28.48" | May 2016 | 20.6 | 8.5 | 12.5 | 2.3 | 0.034 | 7.2 |
| | | Aug. 2016 | 22.2 | 7.8 | 7.5 | 2.3 | 0.013 | 1.4 |
| | | Feb. 2017 | 2.6 | 8.2 | 14.6 | 2.9 | 0.010 | 1.3 |
| N1 | 38°5'14.95" 127°45'41.24 | May 2016 | 13.2 | 7.9 | 12.1 | 1.3 | 0.011 | 1.9 |
| | | Aug. 2016 | 20.3 | 7.7 | 6.5 | 1.3 | 0.006 | 1.7 |
| | | Feb. 2017 | 3.9 | 7.8 | 12.0 | 0.9 | 0.008 | 1.0 |
| N2 | 37°50'5.35" 127°40'41.1" | May 2016 | 17.1 | 7.8 | 13.3 | 1.4 | 0.021 | 5.4 |
| | | Aug. 2016 | 19.7 | 7.2 | 7.5 | 1.7 | 0.013 | 4.7 |
| | | Feb. 2017 | 3.7 | 8.0 | 12.8 | 1.6 | 0.006 | 1.2 |
| N3 | 37°40'28.6" 127°23'2.86" | May 2016 | 16.5 | 6.9 | 9.9 | 1.9 | 0.013 | 3.4 |
| | | Aug. 2016 | 24.5 | 7.2 | 5.8 | 1.6 | 0.008 | 2.2 |
| | | Feb. 2017 | 3.1 | 8.0 | 13.8 | 1.9 | 0.007 | 1.2 |
| H1 | 37°32'29.75" 127°14'35.2 | May 2016 | 17.6 | 8.1 | 10.1 | 2.1 | 0.029 | 7.2 |
| | | Aug. 2016 | 26.1 | 7.8 | 6.0 | 1.8 | 0.019 | 4.1 |
| | | Feb. 2017 | 3.3 | 8.2 | 14.5 | 2.5 | 0.008 | 2.9 |
| H2 | 37°34'31.77" 127°9'38.17" | May 2016 | 19.1 | 8.3 | 11.2 | 3.2 | 0.076 | 4.0 |
| | | Aug. 2016 | 26.2 | 7.9 | 7.0 | 1.9 | 0.017 | 2.1 |
| | | Feb. 2017 | 3.5 | 8.3 | 15.1 | 2.6 | 0.009 | 2.5 |
| H3 | 37°32'2.06" 127°2'15.63" | May 2016 | 21.2 | 8.1 | 9.6 | 2.4 | 0.038 | 4.8 |
| | | Aug. 2016 | 25.5 | 7.6 | 6.4 | 2.8 | 0.038 | 2.9 |
| | | Feb. 2017 | 3.8 | 7.4 | 12.1 | 4.1 | 0.021 | 2.4 |
| H4 | 37°31'31.66" 127°1'0.82" | May 2016 | 20.8 | 7.8 | 9.4 | 2.5 | 0.039 | 6.4 |
| | | Aug. 2016 | 25.5 | 7.4 | 5.3 | 3.0 | 0.053 | 4.0 |
| | | Feb. 2017 | 4.5 | 7.3 | 10.6 | 5.7 | 0.039 | 2.5 |
| H5 | 37°33'0.27" 126°53'46.28" | May 2016 | 21.3 | 7.9 | 9.6 | 3.3 | 0.083 | 6.0 |
| | | Aug. 2016 | 25.6 | 7.5 | 5.6 | 3.5 | 0.109 | 11.6 |
| | | Feb. 2017 | 3.2 | 7.6 | 12.4 | 5.8 | 0.042 | 2.8 |
| H6 | 37°35'44" 126°49'1.46" | May 2016 | 20.3 | 7.3 | 9.0 | 4.7 | 0.108 | 24.2 |
| | | Aug. 2016 | 25.3 | 7.7 | 7.3 | 4.2 | 0.127 | 46.0 |
| | | Feb. 2017 | 3.6 | 7.7 | 13.1 | 7.5 | 0.046 | 40.8 |
| H7 | 37°39'6.73" 126°43'17.93" | May 2016 | 23.8 | 7.6 | 9.9 | 4.5 | 0.352 | 20.0 |
| | | Aug. 2016 | 24.3 | 7.7 | 6.2 | 4.2 | 0.190 | 76.3 |
| | | Feb. 2017 | 3.9 | 7.5 | 11.9 | 8.5 | 0.097 | 499.0 |

**Table S2.** Representative fecal OTUs selected from the OTU table of the Earth Microbiome Project.

| Rank | OTU | Median abundance in human distal gut samples (%) | Frequency in human distal gut samples (%) | Frequency in non-human samples (%) | Taxonomy (phylum; class; order; family; genus) | Median abundance of summed OTUs from top to the current rank in human distal gut (%) |
|---|---|---|---|---|---|---|
| 1 | otu_124280 | 3.4 | 94.2 | 17.1 | Firmicutes; *Clostridia*; *Clostridiales*; *Peptostreptococcaceae*; - | 3.2 |
| 2 | otu_88633 | 3.2 | 100.0 | 5.3 | Firmicutes; *Clostridia*; *Clostridiales*; *Lachnospiraceae*; *Blautia* | 9.0 |
| 3 | otu_190063 | 2.8 | 91.9 | 3.5 | Actinobacteria; *Actinobacteria*; *Bifidobacteriales*; *Bifidobacteriaceae*; *Bifidobacterium* | 12.9 |
| 4 | otu_139096 | 2.4 | 98.8 | 3.8 | Firmicutes; *Clostridia*; *Clostridiales*; *Lachnospiraceae*; *Blautia* | 16.3 |
| 5 | otu_101273 | 2.1 | 93.0 | 2.4 | Firmicutes; *Clostridia*; *Clostridiales*; *Lachnospiraceae*; *Coprococcus* | 18.4 |
| 6 | otu_157280 | 2.0 | 90.7 | 5.2 | Firmicutes; *Clostridia*; *Clostridiales*; *Lachnospiraceae*; - | 21.9 |
| 7 | otu_55941 | 1.5 | 96.5 | 2.8 | Firmicutes; *Clostridia*; *Clostridiales*; *Ruminococcaceae*; - | 22.9 |
| 8 | otu_190980 | 1.4 | 93.0 | 3.1 | Firmicutes; *Clostridia*; *Clostridiales*; *Lachnospiraceae*; - | 26.3 |
| 9 | otu_196163 | 1.3 | 98.8 | 3.1 | Firmicutes; *Clostridia*; *Clostridiales*; *Lachnospiraceae*; *Coprococcus* | 26.7 |
| 10 | otu_114748 | 1.0 | 89.5 | 2.8 | Firmicutes; *Erysipelotrichi*; *Erysipelotrichales*; Erysipelotrichaceae; - | 28.8 |
| 11 | otu_106711 | 0.8 | 95.3 | 3.7 | Firmicutes; *Clostridia*; *Clostridiales*; *Ruminococcaceae*; - | 30.0 |
| 12 | otu_163574 | 0.8 | 90.7 | 1.4 | Firmicutes; *Clostridia*; *Clostridiales*; *Peptostreptococcaceae*; - | 31.8 |
| 13 | otu_190162 | 0.7 | 86.0 | 1.5 | Firmicutes; *Clostridia*; *Clostridiales*; *Lachnospiraceae*; *Dorea* | 32.2 |
| 14 | otu_86215 | 0.7 | 93.0 | 9.3 | Firmicutes; *Bacilli*; *Lactobacillales*; *Streptococcaceae*; *Streptococcus* | 34.4 |
| 15 | otu_1911 | 0.6 | 74.4 | 1.8 | Actinobacteria; *Coriobacteriia*; *Coriobacteriales*; *Coriobacteriaceae*; *Collinsella* | 35.5 |
| 16 | otu_38125 | 0.6 | 90.7 | 5.2 | Firmicutes; *Clostridia*; *Clostridiales*; *Ruminococcaceae*; *Faecalibacterium* | 40.2 |
| 17 | otu_138262 | 0.5 | 96.5 | 2.3 | Firmicutes; *Clostridia*; *Clostridiales*; *Lachnospiraceae*; - | 42.7 |
| 18 | otu_156190 | 0.5 | 97.7 | 2.5 | Firmicutes; *Clostridia*; *Clostridiales*; *Lachnospiraceae*; *Blautia* | 43.2 |
| 19 | otu_85022 | 0.5 | 87.2 | 4.3 | Firmicutes; *Clostridia*; *Clostridiales*; *Clostridiaceae*; - | 44.5 |
| 20 | otu_103043 | 0.4 | 80.2 | 1.9 | Bacteroidetes; *Bacteroidia*; *Bacteroidales*; *Rikenellaceae*; - | 45.3 |
| 21 | otu_69769 | 0.3 | 86.0 | 2.2 | Firmicutes; *Clostridia*; *Clostridiales*; *Lachnospiraceae*; *Ruminococcus* | 47.7 |
| 22 | otu_95259 | 0.3 | 95.3 | 1.2 | Firmicutes; *Clostridia*; *Clostridiales*; *Lachnospiraceae*; *Dorea* | 48.2 |
| 23 | otu_152968 | 0.3 | 86.0 | 1.2 | Firmicutes; *Clostridia*; *Clostridiales*; *Lachnospiraceae*; *Blautia* | 48.3 |
| 24 | otu_148432 | 0.2 | 76.7 | 1.7 | Firmicutes; *Clostridia*; *Clostridiales*; *Clostridiaceae*; - | 49.1 |
| 25 | otu_48195 | 0.2 | 81.4 | 1.4 | Firmicutes; *Clostridia*; *Clostridiales*; *Lachnospiraceae*; *Coprococcus* | 49.4 |
| 26 | otu_2274 | 0.2 | 79.1 | 1.6 | Firmicutes; *Clostridia*; *Clostridiales*; *Clostridiaceae*; *Clostridium* | 49.4 |
| 27 | otu_83279 | 0.1 | 69.8 | 2.4 | Bacteroidetes; *Bacteroidia*; *Bacteroidales*; *Bacteroidaceae*; *Bacteroides* | 51.5 |
